# Mesenchymal Stem Cells Ameliorated Glucolipotoxicity in HUVECs through TSG-6

**DOI:** 10.3390/ijms17040483

**Published:** 2016-04-01

**Authors:** Xingxing An, Lan Li, Younan Chen, Ai Luo, Zuyao Ni, Jingping Liu, Yujia Yuan, Meimei Shi, Bo Chen, Dan Long, Jingqiu Cheng, Yanrong Lu

**Affiliations:** 1 Key Laboratory of Transplant Engineering and Immunology, Ministry of Health; West China Hospital, Sichuan University, Chengdu 610041, China; idealangelan@163.com (X.A.); findingclover@sohu.com (L.L.); chenyounan@scu.edu.cn (Y.C.); liujingping@scu.edu.cn (J.L.); yimianfenzi@163.com (Y.Y.); smmcuco@hotmail.com (M.S.); cb0402022@sina.com (B.C.); longd37@126.com (D.L.); 2 School of Biomedical Sciences, CHIRI Biosciences, Curtin University, GPO Box U1987, Perth, WA 6845, Australia; 3 Sichuan Neo-Life Stem Cell Biotech Inc. Chengdu 610041, China; luoai1239@sina.com.cn; 4 Donnelly Centre for Cellular and Biomolecular Research, University of Toronto, Toronto, ON M5S 3E1, Canada; zyni8@hotmail.com

**Keywords:** HUVECs, MSCs, glucolipotoxicity, inflammation, tumor necrosis factor-α stimulated protein 6 (TSG-6)

## Abstract

Glucolipotoxicity is one of the critical causal factors of diabetic complications. Whether mesenchymal stem cells (MSCs) have effects on glucolipotoxicity in human umbilical vein endothelial cells (HUVECs) and mechanisms involved are unclear. Thirty mM glucose plus 100 μM palmitic acid was used to induce glucolipotoxicity in HUVECs. MSCs and HUVECs were co-cultured at the ratio of 1:5 via Transwell system. The mRNA expressions of inflammatory factors were detected by RT-qPCR. The productions of reactive oxygen species (ROS), cell cycle and apoptosis were analyzed by flow cytometry. The tumor necrosis factor-α stimulated protein 6 (TSG-6) was knockdown in MSCs by RNA interference. High glucose and palmitic acid remarkably impaired cell viability and tube formation capacity, as well as increased the mRNA expression of inflammatory factors, ROS levels, and cell apoptosis in HUVECs. MSC co-cultivation ameliorated these detrimental effects in HUVECs, but no effect on ROS production. Moreover, TSG-6 was dramatically up-regulated by high glucose and fatty acid stimulation in both MSCs and HUVECs. TSG-6 knockdown partially abolished the protection mediated by MSCs. MSCs had protective effects on high glucose and palmitic acid induced glucolipotoxicity in HUVECs, and TSG-6 secreted by MSCs was likely to play an important role in this process.

## 1. Introduction

Diabetes is one of the major chronic diseases that is alarmingly increasing burdens for public health around the world. Hyperglycemia and hyperlipidemia are important characteristics of diabetes, and are critical causal factors of diabetic complications [1,2]. Diabetic vascular diseases, including large blood vessels injuries and microvascular lesions are responsible to the dominant morbidity and mortality in diabetes, which are closely related to the glucotoxicity and lipotoxicity occurred after diabetes onset [3]. Furthermore, endothelial dysfunction is a hallmark of diabetic vascular diseases, observed in the preclinical stage of diabetes [4]. Extensive studies have disclosed that several mechanisms contribute to the impairment of endothelial function. In regard to diabetes, hyperglycemia and hyperlipidemia are driving factors for the chronic inflammation and excessive reactive oxygen species, mainly superoxide in the body, resulting in apoptosis of endothelial cells and imbalance of nitric oxide (NO) production, the most important regulator of endothelial function [5].

Palmitic acid is the most abundant saturated free fatty acids in the body, and accounts for approximately 26% of the total content of free fatty acids in plasma [6]. Excessive palmitic acid uptake by fat-diet participates in oxidative stress, inflammatory reaction, endoplasmic reticulum (ER) stress, and mitochondrial dysfunction, contributing to insulin resistance and endothelial cells dysfunction as well [7]. Epidemiologically, obesity is a main cause of type 2 diabetes (T2DM), however, not all the obese patients will develop T2DM in their life time. Based on this phenomena, the theory of “glucolipotoxicity” is proposed, that means glucotoxicity and lipotoxicity are interacted rather than separate adverse forces on the metabolic cells and tissues. It is hypothesized that lipids over load induced lipotoxicity is depended on the context of chronic hyperglycemia [8].

Mesenchymal stem cells (MSCs) have multi-directional differentiation potential, and can be obtained from bone marrow, adipose tissue, umbilical cord blood, skin, tendon, muscle, and dental pulp *etc.* [9,10,11]. Plenty of evidence has demonstrated that MSCs are potent immune modulators, which allows them attractive for therapy of inflammatory diseases [12]. Paracrine of a broad range of trophic factors or immune regulators has been considered as the primary mechanism of MSCs mediated protective effects observed in animal models of diabetic nephropathy, peripheral arterial diseases and ischemia, highlighting their capability to promote vascular regeneration [13]. Preliminary evidence showed that MSCs transplantation may be effective for T2DM. Patients receiving autologous MSCs in islet transplantation for one year showed improved metabolisms and reduced insulin demand [14]. In our previous study in diabetic nephropathy on rhesus monkey, we observed that MSCs reduced inflammatory factors and chemokines in kidney, ameliorated kidney injuries and improved renal function (data unpublished) [15]. However, whether MSCs are able to protect glucolipotoxicity in endothelial cells and the underlying mechanisms are still elusive.

In the present study, we were aiming to explore the protective effects of MSCs on high glucose and high palmitic acid induced glucolipotoxicity in human umbilical vein endothelial cells (HUVECs), and reveal the relevant molecular mechanisms. Given that the tumor necrosis factor-α (TNF-α)-stimulated protein 6 (TSG-6) plays an important role in protection of inflammation, we used siRNA targeting TSG-6 in MSCs to investigate the role of TSG-6 in MSCs mediated amelioration of glucolipotoxicity in endothelial dysfunction.

## 2. Results

### 2.1. High Glucose and High Palmitic Acid Induced Inflammation and Cell Dysfunction in Human Umbilical Vein Endothelial Cells (HUVECs)

**Figure 1:**
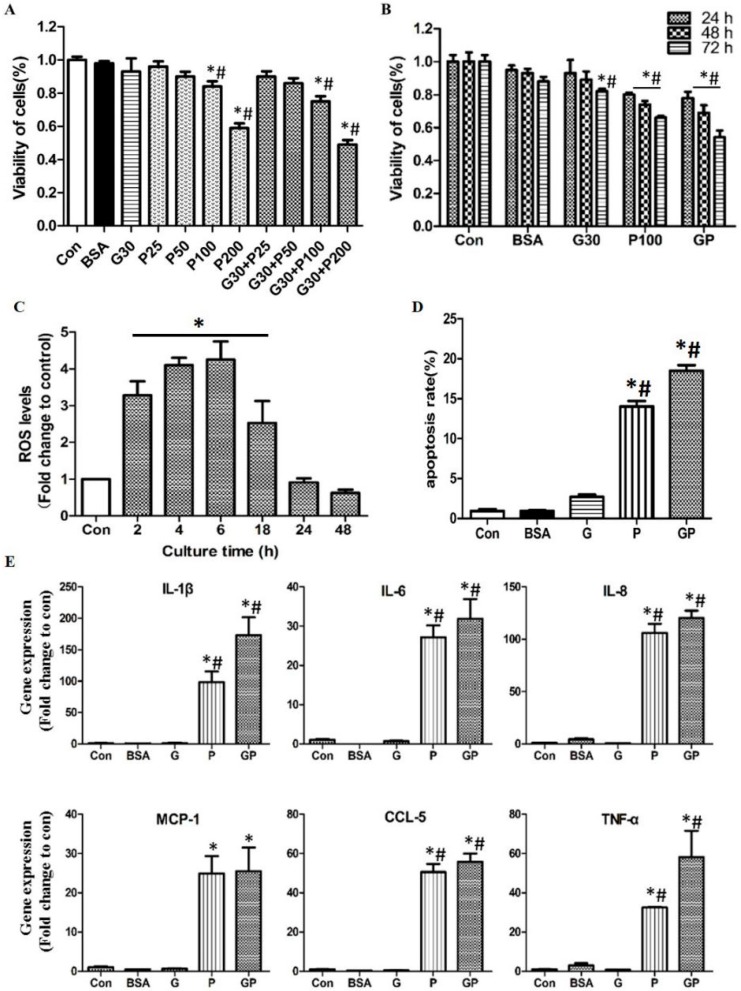
The effects of high glucose and palmitic acid on cell viability, reactive oxygen species (ROS) production, cell apoptosis and inflammation in human umbilical vein endothelial cells (HUVECs). (**A**) Dose dependent impairment of cell viability by 24 h palmitic acid (P) and glucose (G) treatments; (**B**) Time dependent impairment of cell viability by 24–72 h treatments of G or/and P. Cell viability was determined by CCK-8 kit; (**C**) ROS levels after GP treatment for 2–48 h were measured via flow cytometry; (**D**) Cell apoptosis was determined by Annexin-V and PI staining via flow cytometry in 48 h, and Annexin-V and PI double positive staining was calculated as late cell apoptosis rate. All the above data were presented as the percentage of control value; and (**E**) Relative gene expression of inflammation factors such as, IL-1β, IL-6, IL-8, monocyte chemoattractant protein-1 (MCP-1), CC chemokine ligands 5 (CCL-5), and TNF-α by RT-qPCR in 24 h. Gene expression was normalized by β-Actin. Data were present as the mean ± SD for three independent experiments. G, glucose; P, palmitic acid; BSA, bovine serum albumin, as vehicle control; and GP, 30 mM glucose plus 100 μM palmitic acid. * *p* < 0.05 GP, G or P *versus* control, # *p* < 0.05 G or P *versus* GP.

Firstly, we assessed the effects of different concentrations of palmitic acid (P) with or without glucose (G) on the viability of HUVECs. Dose dependence of palmitic acid combined with 30 mM glucose (a widely used concentration of high glucose) induced cellular toxicity was demonstrated after 24 h treatment. The results suggested that glucose combined with palmitic acid (100 and 200 μM) showed the synergistic effect to inhibit the cell viability in HUVECs (Figure 1A). Furthermore, time dependent effect of high glucose and/or high fatty acid was convinced after 24 to 72 h treatment (Figure 1B). Significant alterations were observed in 30 mM glucose plus 100 μM palmitic acid (GP) treatment, showing time dependent impairment of cell viability as 78% ± 3.66% in 24 h, 69% ± 4.45% in 48 h, and 54% ± 4.01% in 72 h, respectively. The morphology changes and intracellular lipid droplets of high glucose and high palmitic acid treated HUVECs were also observed under light microscope (Figure S1). Therefore, the GP treatment for 24 or 48 h was used in further experiments if not addressed individually.

Excessive oxidative stress and apoptosis are critical mechanistic aspects in the development of diabetes. To further verify the detrimental effects of high glucose and high palmitic acid on HUVECs. Reactive oxygen species (ROS) production and cell apoptosis were determined via flow cytometry. After 30 mM glucose plus 100 μM palmitic acid (GP) treatment, the generation of total intercellular ROS showed time dependent changes. From 2 to 6 h, ROS levels were robustly elevated. However, after 18 h, ROS production dropped, and went back to normal level or even less at 24 h (Figure 1C). Cell apoptosis was determined via Annexin-V-FLUOS kit by flow cytometry after 48 h GP treatment (Figure S2). The late stage apoptotic rate (double positive cell population) of GP treated HUVEC was 18.5% ± 0.5% at 48 h, in comparison to 1.5% ± 0.2% in control cells. Additionally, 30 mM glucose treatment displayed much less pro-apoptotic effect than 100 μM palmitic acid, implying that high fatty acid has made the primary contribution of apoptosis in GP treatment. On the other hand, GP showed more apoptosis than P treatment, suggesting that high glucose has synergetic effect on lipotoxicity (Figure 1D).

With regard to inflammation, chronic inflammation plays a crucial role in the mechanism of diabetes and cardiovascular diseases. Herein, we asked whether the *in vitro* HUVECs stimulated by high glucose and high palmitic acid were able to mimic the inflammation in diabetes. After G, P or GP treatment for 24 h, the mRNA expression of inflammatory cytokines (IL-1β, IL-6, IL-8, and TNF-α) and chemokines monocyte chemoattractant protein-1 (MCP-1) and CC chemokine ligands 5 (CCL-5) were dramatically up-regulated in HUVECs (Figure 1E). Interestingly, 30 mM glucose treatment has not shown any pro-inflammatory effect in this scenario, but showing exacerbating effect when combined with 100 μM palmitic acid, which was in line with the apoptosis results described above.

Taken together, the above results indicated that 30 mM glucose combined with 100 μM palmitic acid for 24 or 48 h was effective to cause deterioration in HUVECs, implying that it might be an ideal *in vitro* model to mimic endothelial injury in diabetes.

### 2.2. Protective Effects of Mesenchymal Stem Cells (MSCs) on HUVECs Dysfunction Driven by High Glucose and Palmitic Acid

#### 2.2.1. MSCs Alleviated Growth Arrest and Apoptosis but Not Reactive Oxygen Species (ROS) Production

**Figure 2:**
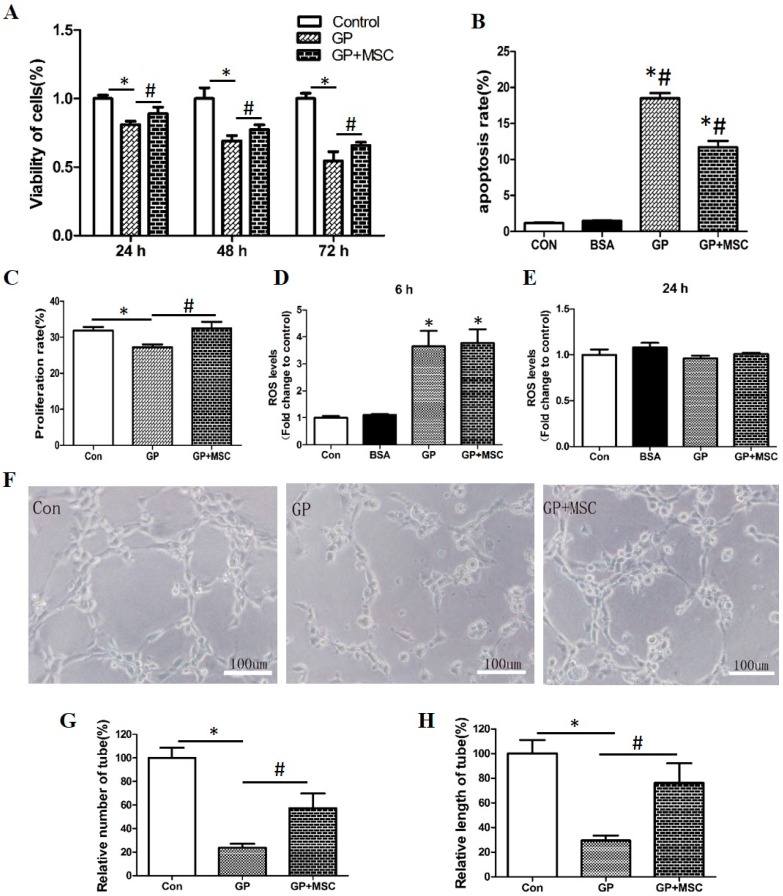
The protective effects of mesenchymal stem cells (MSCs) on GP treated HUVECs. 30 mM glucose plus 100 μM palmitic acid (GP) were used to treat HUVECs with or without MSCs in Transwell system. (**A**) Time dependent cell viability; (**B**) Late stage cell apoptosis; (**C**) Cell proliferation rate was analyzed by cell cycle data. Proliferation rate = (S + G2/M)/(G0/G1 + S + G2/M) × 100%; (**D**,**E**) ROS levels after 6 or 24 h treatment; (**F**) Tube formation ability estimated by inverted light microscope (Scale bar = 100 μm). The average number (**G**) and length (**H**) of complete capillary-like tubes formed by cells were calculated by Image-Pro Plus software. * *p* < 0.05 GP or GP + MSC *versus* control, # *p* < 0.05 GP *versus* GP + MSC.

HUVECs (3 × 10^4^/well) and MSCs (6 × 10^3^/well) were co-cultured via Transwell in 6-well plate. The proper ratio of MSCs to HUVECs were mainly evaluated by cell viability changes (Figure S3), and ratio 1:5 was chosen for the further treatment. Cell viabilities after 24, 48 and 72 h treatment, apoptosis after 48 h, and cell cycles after 48 h treatment of HUVECs were measured, respectively. MSCs intervention restored HUVECs viability from 81.0% ± 2.4% to 89.1% ± 4.7% in 24 h treatment, from 69.2% ± 3.7% to 77.4% ± 3.5% in 48 h, and from 54.5% ± 6.7% to 65.8% ± 2.2% in 72 h, respectively (Figure 2A). Consistently, late stage apoptosis of HUVECs with MSCs intervention (GP + MSC) was significantly attenuated (11.9% ± 0.3%) in comparison to that in GP group (18.5% ± 0.5%) after 48 h (Figure 2B). Additionally, cell proliferation of HUVECs was significantly impaired after 48 h GP treatment, nevertheless, MSCs restored it from 27.2% ± 0.6% to 32.5% ± 1.2% (Figure 2C). However, there was no decrease of ROS level in HUVECs with MSCs intervention after 6 or 24 h GP treatment (Figure 2D,E). These results indicated that the protective effect of MSCs on GP treated HUVECs was not related to the attenuation of oxidative stress.

#### 2.2.2. MSCs Improved Capillary-Like Tube Formation Ability

Tube formation ability is one of the important indicators of HUVECs function. After 48 h GP simulation, the tube formation was undoubtedly deteriorated with the decrease of tube number and length (Figure 2F). Interestingly, capillary-like tube number (Figure 2G) and tube length (Figure 2H) were substantially increased with MSCs intervention.

#### 2.2.3. MSCs Reduced Gene Expression of Inflammatory Cytokines and Disrupted NF-κB Activation

**Figure 3:**
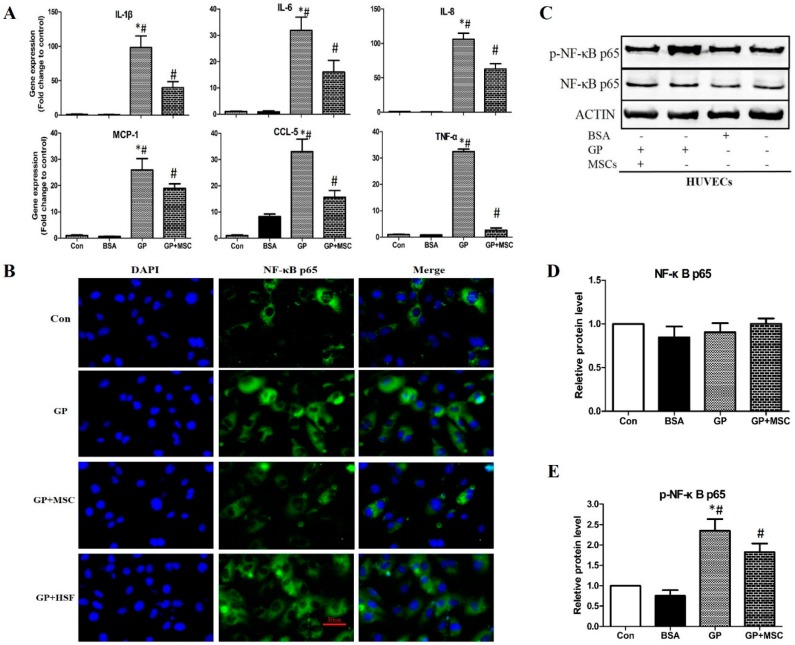
MSCs reduced mRNA expression of inflammatory factors and ameliorated NF-κB activation in HUVECs. (**A**) Gene expression of inflammatory factors after 24 h treatment by RT-qPCR; (**B**) Immunofluorescence of p-NF-κB p65 (green) and 4,6-Diamidino-2-phenylindole (DAPI) (blue) in HUVECs after 24 h treatment (Scale bar = 50 μm); (**C**) The protein level of total or p-NF-κB p65 after 48 h treatment, detected by Western blot; and (**D**,**E**) are quantification of (**C**). * *p* < 0.05 GP *versus* control, # *p* < 0.05 GP *versus* GP + MSC.

As we expected, the dramatic up-regulations of inflammatory cytokines (IL-1β, IL-6, IL-8, and TNF-α) and chemokines (MCP-1 and CCL-5) by high nutrients treatment were significantly abrogated by MSCs intervention, as detected by RT-qPCR (Figure 3A). Protein levels of IL-1β and MCP-1 in the supernatant were also detected by Western blot (Figure S4). Based on the literatures, NF-κB activation plays a central role in inflammation and palmitic acid is able to stimulate the activation of NF-κB signaling in endothelial cells [16]. We further explored whether MSCs could interfere NF-κB activation. The microscopy showed that after exposure to GP for 24 h, phosphorylation of NF-κB p65 was obviously increased, which was supposed to be relevant to the enhanced activation of NF-κB and its downstream pro-inflammatory and pro-apoptotic effects. The increase of phosphor-NF-κB p65 in HUVECs was attenuated with MSCs co-cultivation but not with human skin fibroblasts (HSFs) co-cultivation (Figure 3B). Moreover, the protein expression of NF-κB p65 was determined by Western blot (Figure 3C). Time dependent effect of high glucose and high fatty acid was convinced after 24 to 48 h treatment (Figure S5). After GP treatment for 48 h, no alteration of total NF-κB p65 was found (Figure 3D). However, the phosphorylation of NF-κB p65 (p-NF-κB p65) was notably increased by GP, and this effect was partially abolished by MSCs intervention (Figure 3E), suggesting that MSCs was able to interrupt NF-κB p65 activation and may subsequently ameliorate the downstream pro-inflammation and pro-apoptosis reactions.

### 2.3. The Protective Role of MSCs Was Related to the Secretion of Tumor Necrosis Factor-α Stimulated Protein 6 (TSG-6)

#### 2.3.1. Expression of TSG-6 in High Glucose and Palmitic Acid Stimulated Cells

**Figure 4:**
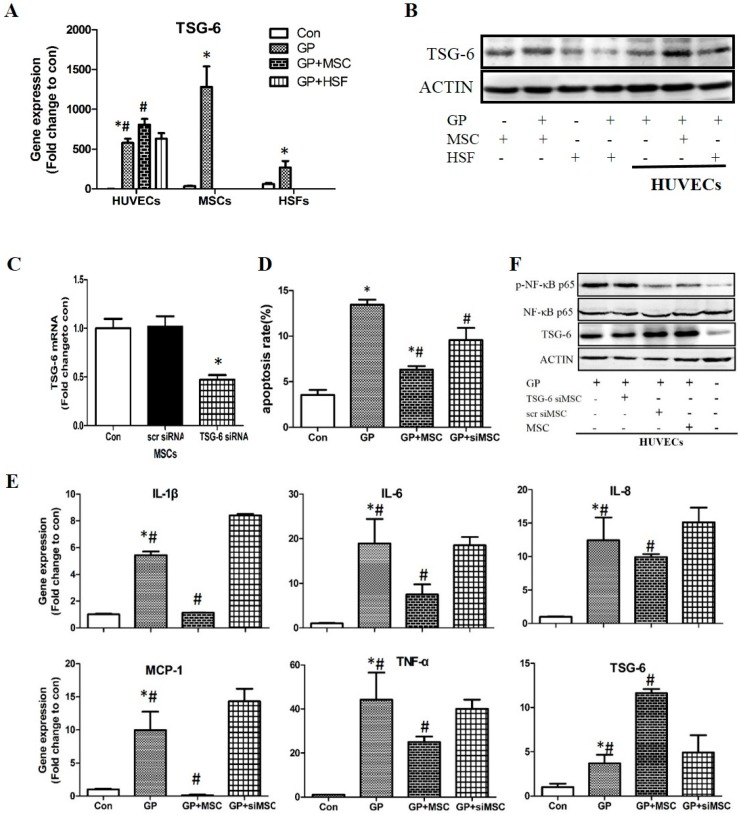
The expression of tumor necrosis factor-α stimulated protein 6 (TSG-6) in high glucose and palmitic acid treated cells and the effects of TSG-6 siRNA transfected MSCs on HUVECs. (**A**) Gene expression of TSG-6 on HUVECs, MSCs, and human skin fibroblasts (HSFs) after 24 h GP treatment; (**B**) protein levels of TSG-6 after 48 h treatment; (**C**) Gene expression of TSG-6 in MSCs after transfected with 50 nM TSG-6 siRNA and 50 nM scrambled siRNA (scr siRNA) as control for 24 h; (**D**) Cell apoptosis of HUVECs after 48 h GP treatment; (**E**) Gene expression of the inflammation factors of HUVECs after 24 h treatment; and (**F**) The protein levels of TSG-6 and p-NF-κB p65 in HUVECs after 48 h treatment. * *p* < 0.05 TSG-6 siRNA *versus* normal control, GP *versus* control, # *p* < 0.05 GP *versus* GP + MSC, GP + MSC *versus* GP + siMSC.

Given that TSG-6 is a potent anti-inflammatory factor [17], we asked whether the protective effect from MSCs was associated with TSG-6. Firstly, mRNA expression of TSG-6 was measured by RT-qPCR after 24 h GP treatment, TSG-6 gene expression was up-regulated in HUVECs, MSCs and HSFs, respectively, whereas, MSCs displayed the highest expression (Figure 4A). Additionally, much higher TSG-6 level was detected in HUVECs co-cultured with MSCs than in absence of MSCs. In consistence, Western blot results showed that TSG-6 protein level was markedly increased in MSCs and HUVECs after 48 h GP treatment (Figure 4B), and much higher expression was found in MSCs than in HSFs. Then, we co-cultured HUVECs with MSCs or HSFs, and found that MSCs significantly increased TSG-6 protein level in HUVECs after GP treatment in comparison to GP only group. However, this effect was not observed in HUVECs co-cultured with HSFs. These results indicated that GP treatment profoundly regulated TSG-6 expression, and MSCs co-cultivation was able to enhance this effect in HUVECs.

#### 2.3.2. The Effects of TSG-6 Knockdown on MSCs and MSCs Co-Cultured HUVECs

To further explore the relevance of TSG-6 with MSCs mediated protection on high glucose and palmitic acid damaged HUVECs, TSG-6 was knockdown in MSCs by TSG-6 siRNA transfection (Figure S6), and siRNA-3 (TSG-6 siRNA below) was chosen for future experiments. RT-qPCR results convinced that TSG-6 siRNA interference decreased 61% ± 2% gene expression of TSG-6 in comparison to the normal control (Figure 4C). We co-cultured HUVECs with TSG-6 siRNA transfected MSCs and found that TSG-6 knockdown in MSCs partially abolished MSCs mediated protective effect on cell apoptosis (9.55% ± 1.35% *versus* 6.30% ± 0.40%, GP + siMSC *versus* GP + MSC) (Figure 4D). Interestingly, TSG-6 knockdown showed striking effect on mRNA expressions of inflammatory factors. As shown previously, MSCs inhibited GP induced inflammatory factors expression, however, TSG-6 knockdown abolished most of this effect, and exhibited approximately similar expression levels with GP group (Figure 4E). Furthermore, knockdown of TSG-6 led to enhancement of NF-κB activation, convinced by increased phosph-NF-κB p65 level in comparison to wildtype MSCs group (Figure 4F). These results suggested that TSG-6 secreted by MSCs might be an important factor to mediate the anti-inflammation effect in HUVECs.

## 3. Discussion

In the present study, we were using high glucose and high saturated fatty acid to induce glucolipotoxicity in endothelial cells, and explored the protective effects of MSCs through paracrine. The toxicity of excessive exogenous nutrients was demonstrated by declines in cell viability and tube formation ability, as well as increases in ROS production, cell apoptosis, and expression of cytokines and chemokines. Concomitant incubation of HUVECs and MSCs via a Transwell system showed that MSCs exerted protection of glucolipotoxicity in HUVECs by improving cell viability, cell proliferation, and tube formation, and repressing cell apoptosis and inflammation. In terms of the implying molecular mechanism, the increased NF-κB activation might play a key role in the glucolipotoxicity, and the anti-inflammatory factor TNF-α-induced protein 6 (TSG-6) secreted by MSCs might be associated with MSCs mediated amelioration of inflammation and cell dysfunction in HUVECs, which was evidenced by knockdown of TSG-6 in MSCs.

A great number of literatures addressed gluotoxicity or lipotoxicity in clinical and experimental studies, which is closely associated with the development of diabetes and diabetic cardiovascular diseases. It is worthwhile to note that, high glucose is able to synergetically exacerbate the cellular dysfunction caused by lipotoxicity in the context of diabetes. It is well established in pancreatic β-cell that in the fasting state, fatty acids are primarily transferred into mitochondria for β-oxidation [18]. This is rapidly reversed upon carbohydrate uptake. In high glucose context, glucose is fluxed into β-cells and is metabolized to pyruvate through glycolysis, then enters mitochondria to generate energy. This promotes the formation of citrate that inhibits carnitine palmitoyltransferase-1 (CPT-1) to transport long-chain fatty acyl-coenzyme As (CoAs) into mitochondria for β-oxidation [19]. The accumulated metabolites derived from fatty acid esterification impair β-cell function. Ceramide synthesized from long chain (LC)-CoA and serine may serve as an important mediator of free fatty acid (FFA)-induced cell death [20]. Given that, we treated HUVECs with the combination of high glucose (30 mM) and high long-chain fatty acid (100 μM palmitate) to mimic the typical glucolipotoxicity in diabetic patients. Agreeable with the hypothesis that glucolipotoxicity exacerbates endothelial dysfunction, our results showed that GP treatment induced higher toxicity than G30 or P100 treatments, displaying lower cell viability (see Figure 1B) and higher inflammatory factor expression (see Figure 1E). On the other hand, our findings suggested that in 24 or 48 h treatment, palmitic acid contributed much more to the cell damage than glucose, and 30 mM glucose did not show obvious toxicity to the endothelial cells. This was in line with our previous study, which found 30 mM glucose exhibited toxicity after more than 72 h treatment (unpublished data) [21]. Therefore, in the present study, our main aim was to explore the fatty acids induced toxicity to endothelial cells in the context of hyperglycemia.

Previous studies show that the mechanisms of palmitic acid induced cell damages mainly involve insulin resistance [22], endoplasmic reticulum stress (ERS) [23], oxidative stress [24], apoptosis [25,26,27,28,29], autophagy [30], and production of many pro-inflammatory factors [31,32] to cause endothelial dysfunction and cardiovascular diseases. A growing number of researches suggest that low grade of systemic inflammation is an important characteristic of the pathogenesis of T2DM and cardiovascular diseases. A pro-inflammatory shift in vascular gene expression profile, including an upregulation of inflammatory cytokines, chemokines, and adhesion molecules was observed in endothelial aging, which contributes to a pro-inflammatory environment and facilitates both the development of endothelial apoptosis and cell dysfunction. Herein, we found the remarkable alterations of gene expression profile of inflammatory cytokines and chemokines after high glucose and high fatty acids stimulation in HUVECs, consistent to the literatures and our previous studies in diabetic nephropathy (unpublished data) [15]. Moreover, the enhanced expression of phosphorylated NF-κB was demonstrated here, which was also in agreement with other reports that chronic activation of NF-κB is likely responsible for the development of atherosclerosis [33]. MSCs are well-known as immuno-regulator, and their anti-inflammation role has been established *in vitro* and *in vivo*. As we expected, MSCs rescued the cells from glucolipotoxicity driving cell death and cell dysfunction. The alleviation of inflammation by MSCs, showing as decreased expression of cytokines and chemokines and obviously disturbed NF-κB activation, was in line with its anti-inflammatory role in a variety of scenarios. However, it is unexpected that MSCs did not impact ROS production in HUVECs, which was discrepancy to our study of high glucose induced endothelial dysfunction, showing MSCs was able to attenuate mitochondrial ROS. The possible interpretation is that high glucose increases ROS production after a relative long term treatment (72 h), and MSCs exerted anti-oxidant effect also in a time dependent manner. However, in this study, the glucolipotoxicity presented acute increase of ROS production from 2 to 6 h, but no effect after 24 h. It suggested the dynamics between glucotoxicity and lipotoxicity induced oxidative stress are different, and MSCs did not show acute impact on ROS. The detailed mechanism involved is required to be further investigated.

To date, the specific mechanism of MSCs elicited anti-inflammatory response is not fully understood yet. Intensive researches focused on the secretomes of MSCs by paracrine, including a broad spectrum of growth factors, cytokines and chemokines. Nevertheless, which factor plays a key role is still controversial. TSG-6 is identified in the last decade as an anti-inflammatory factor released by a variety of cells in response to a variety of stimuli. As a multifunctional protein, TSG-6 is up-regulated in many physiological and pathological contexts, where it plays critical roles in inflammation and tissue remodeling [34,35]. In transgenic mice overexpressing TSG-6, inflammation reaction was reduced [36]. Conversely, in TSG-6 knockout mice, inflammation was exacerbated [37]. Recently, a negative feed-back loop elicited by MSCs secreted TSG-6 was highlighted in MSCs related immune-modulation. The interpretation is that MSCs are activated by proinflammatory stimuli, and secrete TSG-6 that interacts with CD44 on resident macrophages to decrease Toll-like receptor (TLR)/NF-κB signaling [38]. Herein, we reported at the first time that MSCs attenuate inflammation in endothelial cells which is associated with TSG-6. Our results clearly showed that high glucose and high fatty acids induced robustly increase of TSG-6 expression in MSCs, and meanwhile MSCs enhanced the TSG-6 expression in HUVECs. Gene knockdown of TSG-6 in MSCs partially inhibited the MSCs mediated protection of glucolipotoxicity. Interestingly, the most substantial effects of TSG-6 knockdown were observed on the increase of expression of pro-inflammatory factors as well as activated NF-κB, which is agreeable with the previous reports. In the future study, we plan to investigate the effect of MSCs and TSG-6 on more cell types like arterial and capillary endothelial cells. A better understanding of the molecular mechanisms involved is warranted in the future studies.

In summary, our study clarified the protective effects of MSCs on high glucose and high palmitic acid impaired vascular endothelial cells, and found that TSG-6 was an important factor in MSCs mediated anti-inflammatory response.

## 4. Experimental Section

### 4.1. Materials

Glucose powder (Sigma, St. Louis, MO, USA) was dissolved in sterile PBS. Palmitic acid was prepared according to the protocol modified from previous report [39]. Briefly, palmitic acid (Sigma) was dissolved in 100% ethanol at 70 °C to make a stock solution of 100 mM, then conjugated with 10% fatty acid free-bovine serum albumin (BSA) (Roche Diagnostics, Mannheim, Germany) with volume ratio 1:10 at 55 °C for 10 min to reach 10 mM solution. The concomitant treatment in cell culture with high glucose and high palmitic acid referred to 30 mM glucose plus 100 μM palmitic acid (GP), if not addressed individually. Vehicle control (BSA) was used in all experiments with the same concentration of BSA and ethanol with GP treatment.

### 4.2. Cell Cultures

Human umbilical vein endothelial cells (HUVECs) were isolated from healthy and fresh human umbilical cords via 0.1% collagenase I (Gibco, Grand Island, NY, USA) and cultured in EndoGRO-VEGF Complete Culture Media (Millipore, Bedford, MA, USA). Human umbilical MSCs were provided by SiChuan Neo-Life Stem Cell Biotech Inc. (Chengdu, China) and maintained in Low-glucose dulbecco’s modified eagle medium (DMEM) containing 10% fetal bovine serum (Gibco). Human skin fibroblasts (HSFs) were obtained from prepuce tissue explants. Passage 2 to 3 HUVECs, MSCs and HSFs were used for future experiments. Six-Well Millicell hanging cell culture inserts (Transwells) (Millipore) were used for separating HUVECs with MSCs or HSFs during the co-culture. HUVECs were in the lower chamber, the number ratio of MSCs or HSFs to HUVECs was 1:5. The culture medium for the co-culture is EndoGRO-VEGF Complete Culture Media.

### 4.3. Assessment of Cell Viability

To determine glucose and palmitic acid induced cytotoxicity, HUVECs were seeded at a concentration of 3 × 10^3^ cells/100 μL of culture media into the wells of 96-well plate, after cells confluence reaching 80%, cells were exposed to different concentrations of palmitic acid (25~200 μM) with or without high glucose (30 mM). Time dependent effect was assessed as well by setting the treatment from 24 to 72 h. Cell viability was determined by CCK-8 kit (DOJINDO, CK04, Shanghai, China), the absorbance was detected by microplate reader (BioTek, uQuant, Santa Barbara, CA, USA) at a wavelength of 450 nm.

### 4.4. Flow Cytometry Analysis of ROS Production, Cell Cycle, and Apoptosis

HUVECs with or without co-culture of MSCs were cultured 3 × 10^4^ cells/well in 6-well plate, after cells confluence reaching 80%, cells were treated by GP for a series of time. Intracellular ROS levels were determined by DCFH-DA probe (Beyotime Biotechnology, Shanghai, China) via flow cytometry (Backman Coulter, FC500, Fullerton, CA, USA). Cells were fixed with 70% precooling alcohol at 4 °C for 48 h. After incubation with RNase (100 μg/mL) and propidium iodide (50 μg/mL) for 30 min, cell cycle was measured by flow cytometry. Cell apoptosis was also analyzed via flow cytometry by Annexin-V-FLUOS Staining Kit (Roche) following the manufacture’s protocol.

### 4.5. Total RNA Extraction and Real-Time qPCR

MSCs, HSFs and HUVECs were stimulated with 30 mM glucose and 100 μM palmitic acid for 24 h. Then cell (approximately 3 × 10^5^ cells per sample) RNA was extracted via Tripure Isolation Reagent (Roche). The cDNA was synthesized by reverse transcription using Transcriptor First Strand cDNA Synthesis Kit (Roche). AceQTM qPCR SYBR Green Master Mix (Vazyme biotech, Nanjing, China) was used for qPCR reaction. The relative mRNA expression of inflammation factors and chemokines were determined by qPCR, and β-Actin was used as internal reference gene. Primers were synthesized by Sangon Biotech (Shanghai, China), and their sequences were listed in Table 1.

### 4.6. Immunofluorescence Imaging

The expression of nuclear factor-κB (NF-κB) p65 in HUVECs was detected by immunofluorescence. Briefly, HUVECs with or without co-culture of MSCs or HSFs were stimulated by GP for 24 h. Cells were fixed by 4% paraformaldehyde and then permeabilized with 0.3% Triton. After PBS wash, anti-phospho-NF-κB p65 antibody (1:200, CST, Danvers, MA, USA) was incubated with cells at 4 °C overnight. Goat anti-rabbit IgG conjugated with Alexa Fluor 488 (1:500, Invitrogen, Carlsbad, CA, USA) was used as secondary antibody, and was incubated with cells at 37 °C for 1 h. Nuclei were stained by DAPI (1 μg/mL) for 5~10 min. Images were taken by Leica fluorescent microscope (LEICA, Heidelberg, Germany).

### 4.7. Endothelial Cell Capillary-Like Tube Formation Assay

After 48 h GP treatment, 3 × 10^3^ HUVECs /well were seeded in Matrigel basement membrane matrix (BD Biosciences, San Jose, CA, USA) coated 96-well plate (50 μL/well), and incubated for a further 2~4 h. Tubes were observed by inverted light microscope and four representative fields were analyzed. The average length and number of complete capillary-like tubes formed by cells were calculated by Image-Pro Plus software (Media Cybernetics, Silver Springs, MD, USA).

### 4.8. Small Interfering RNAs (siRNAs) Transfection of MSCs

TNFAIP6 (TSG-6) siRNAs were purchased from RIBOBIO Co., Ltd. (ribo, Guangzhou, China) and the sequences were listed in Table 2. Transient transfection of MSCs was carried out using C10511-05 riboFect™ CP Transfection Kit (166T, ribo) according to the manufacturer’s instructions. Briefly, 3 × 10^4^ MSCs/well were cultured in 24-well plate, each group had three reduplicative wells, after cells confluence reaching 50%, transfection were begun. Diluted 1.25 μL TSG6 siRNAs, NControl (scr siRNA), and si-h-GAPDH with 30 μL 1× Buffer, respectively. Then incubated with 3 μL Reagent at room temperature for 15 min, the final culture medium of each well was 500 μL and the work concentration of siRNA was 50 nM. After transfection for 24 h, cell RNA was extracted and TSG-6 gene expression was detected by RT-qPCR in TSG-6 siRNAs and NControl groups, GAPDH gene expression was tested in si-h-GAPDH group, β-Actin was internal reference. When TSG-6 decreased 60%~80% in genetic level, transfection was proved successful, then transfected MSCs were subjected to further experiments.

### 4.9. Western Blot Analysis

Total cell (approximately 1 × 10^6^ cells per sample) protein was extracted by lysis buffer (RIPA) (Beyotime) containing phenylmethylsulphonyl fluoride (PMSF). Protein concentration was determined via BCA kit (CWBIO, Beijing, China). The expression levels of NF-κB p65, phospho-NF-κB p65, and TSG-6 were determined by SDS-PAGE and immunoblot assays using primary rabbit antibodies for NF-κB p65 (1:1000, AR, Hangzhou, China), phospho-NF-κB p65 (1:1000, CST), and TSG-6 (1:1000, R&D, Minneapolis, MN, USA), and goat anti-rabbit secondary antibody (1:2000). Quantification of protein bands was performed by Image J software (NIH, Bethesda, MD, USA).

### 4.10. Statistical Analysis

Data were presented as mean ± SD. ANOVA was used for intergroup comparison, and a two-sided *p* < 0.05 was considered statistically significant.

## Figures and Tables

**Table 1:** The list of primers sequences.

Gene	Forward Primer (5′ to 3′)	Reverse Primer (5′ to 3′)
IL-1β	GCCGTGTCAGTTGTTGTAGC	TGAAGGGAATCAAGGTGCTC
IL-6	TACATCCTCGACGGCATCTC	GCCATCTTTGGAAGGTTCAG
IL-8	ACTCCAAACCTTTCCACCC	AACTTCTCCACAACCCTCTGC
MCP-1	AGCCAGATGCAATCAATGCC	GGGTCAGCACAGATCTCCTT
CCL-5	CCTGCTGCTTTGCCTACATT	GCACACACTTGGCGATTCT
TNF-α	CTGCCTGCTGCACTTTGGA	TTGAAGAGGACCTGGGAGTAGAT
TSG-6	TGGCTTTGTGGGAAGATACTGT	TGGAAACCTCCAGCTGTCAC
GAPDH	GAACGGGAAGCTCACTGG	GCCTGCTTCACCACCTTCT
β-Actin	CCACGAAACTACCTTCAACTCC	GTGATCTCCTTCTGCATCCTGT

**Table 2:** The list of small interfering RNAs sequences.

Gene	Target	Forward (5′ to 3′)	Reverse (5′ to 3′)
si-h-TNFAIP6001	GCAAATACAAGCTCACCTA	GCAAAUACAAGCUCACCUA	dTdT
		dTdT	CGUUUAUGUUCGAGUGGAU
si-h-TNFAIP6002	GCTACAACCCACACGCAAA	GCUACAACCCACACGCAAA	dTdT
		dTdT	CGAUGUUGGGUGUGCGUUU
si-h-TNFAIP6003	CCAGGTTGCTTGGCTGATT	CCAGGUUGCUUGGCUGAUU	dTdT
		dTdT	GGUCCAACGAACCGACUAA

## Supplementary Materials

Supplementary materials can be found at http://www.mdpi.com/1422-0067/17/4/483/s1.
