# Supplementary material for: Mesenchymal Stem Cells Ameliorated Glucolipotoxicity in HUVECs through TSG-6
Source: Int J Mol Sci. 2016 Apr 1;17(4):483. doi: 10.3390/ijms17040483 (PMC4848939; doi:10.3390/ijms17040483)
Supplement: Supplementary file 1 [file ijms-17-00483-s001.pdf]

Supplementary Materials: Mesenchymal Stem Cells Ameliorated Glucolipotoxicity in HUVECs through TSG-6

Xingxing An, Lan Li, Younan Chen, Ai Luo, Zuyao Ni, Jingping Liu, Yujia Yuan, Meimei Shi, Bo Chen, Dan Long, Jingqiu Cheng and Yanrong Lu

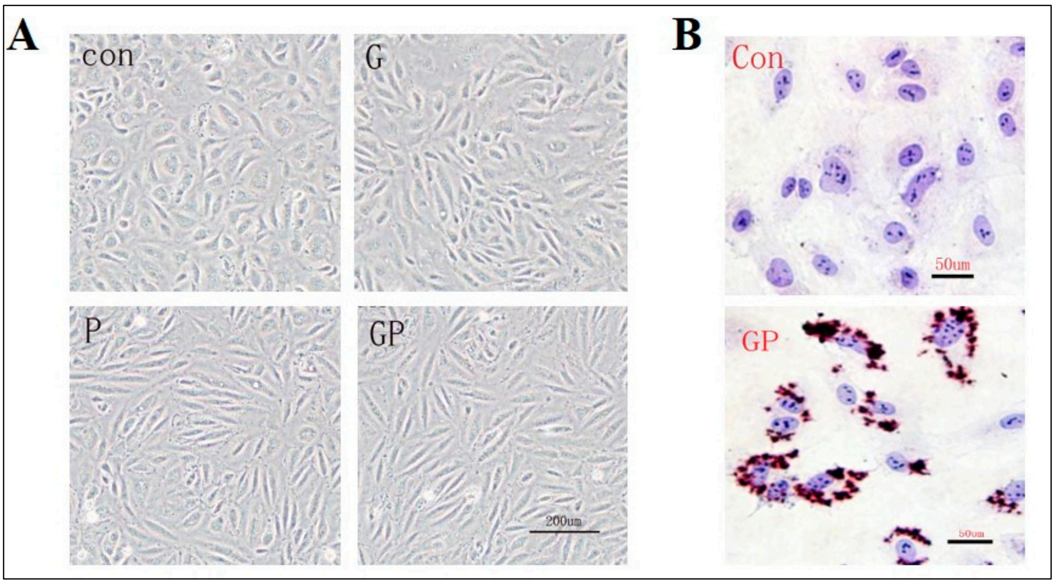

Figure S1. The morphology changes (**A**) and intracellular lipid droplets (**B**) of high glucose and palmitic acid treated HUVECs in 24 h.

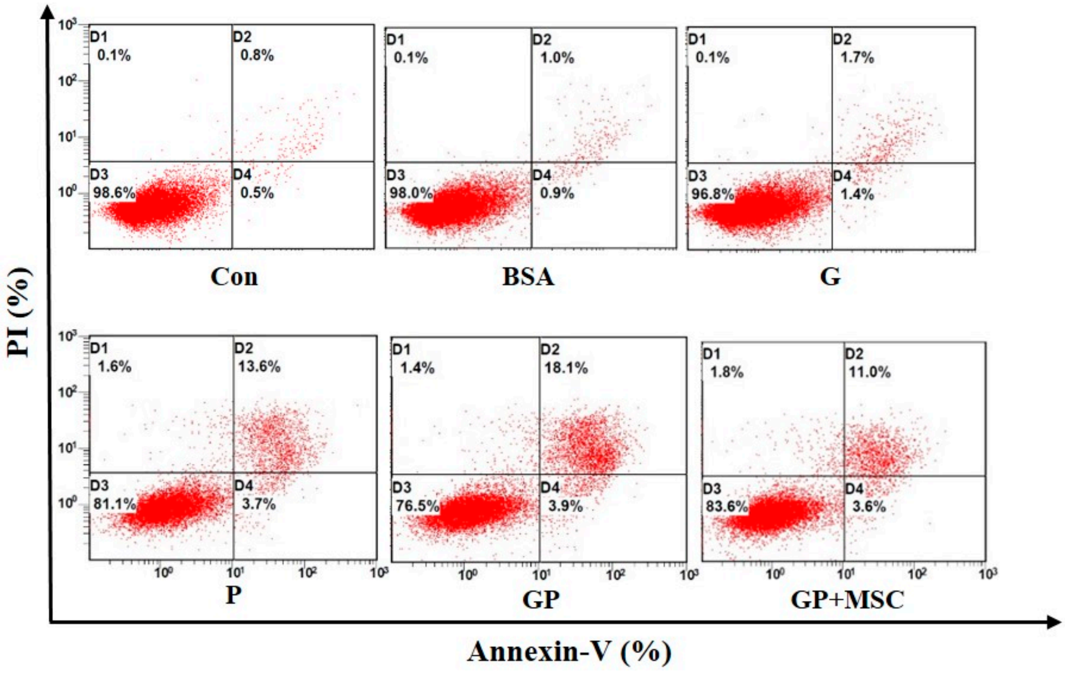

Figure S2. High glucose and palmitic acid induced apoptosis of HUVECs in 48 h. PI: propidium iodide.

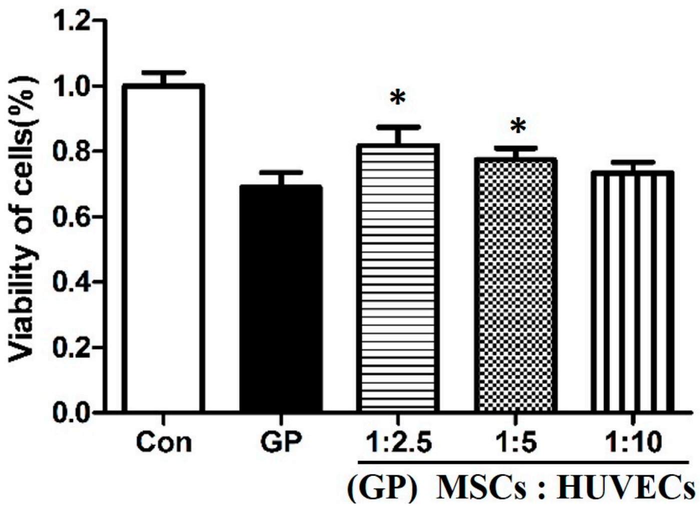

Figure S3. The effect of different ratios of MSCs to HUVECs on cell viability in 48 h. * $p < 0.05$ (GP) MSCs: HUVECs = 1:2.5 or 1:5 *versus* (GP) HUVECs.

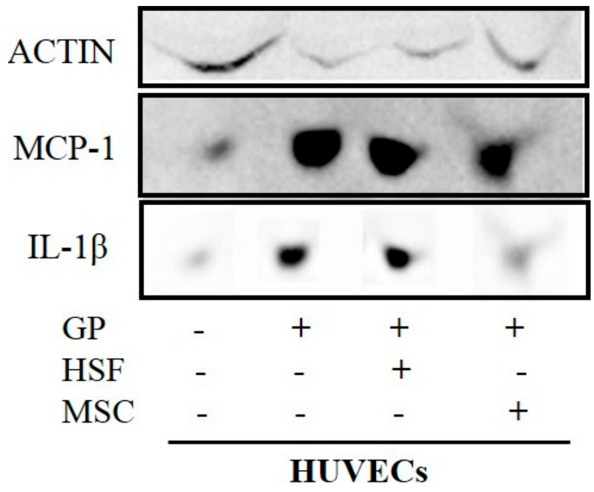

Figure S4. The effect of MSCs on inflammation factors protein levels in 48 h.

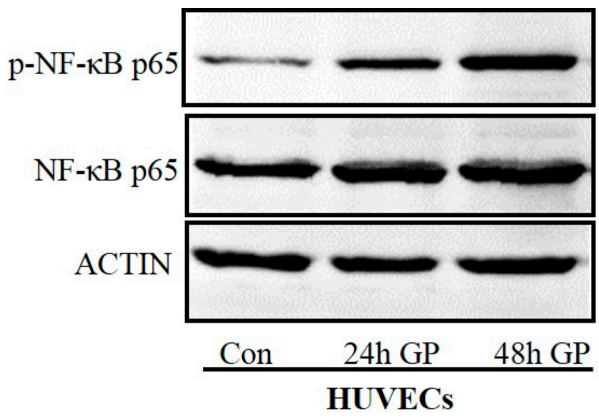

Figure S5. High glucose and palmitic acid increased phosphorylation of NF-κB p65 (p-NF-κB p65) in HUVECs.

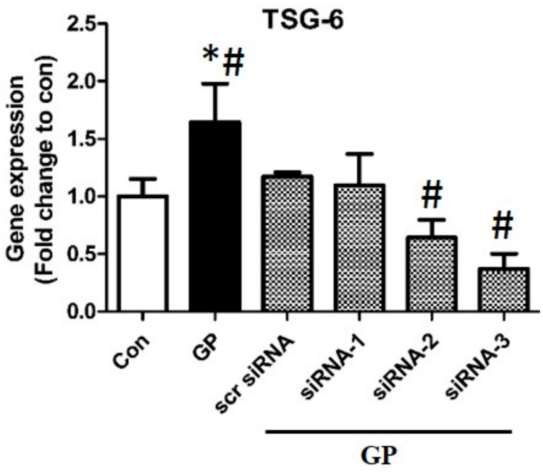

Figure S6. TSG-6 small interfering RNAs (siRNAs) transfection of MSCs. * $p < 0.05$ GP *versus* Con, # $p < 0.05$ GP *versus* siRNA-2(GP) or siRNA-3(GP).
